# Teaching humanities in UK medical schools: towards community-building and coherence

**DOI:** 10.1007/s40592-025-00249-y

**Published:** 2025-05-28

**Authors:** Richard T. Bellis, Sally Frampton, Gina Hadley, Jennifer Wallis, Alan Bleakley, Gabriele De Luca

**Affiliations:** 1 University of St Andrews, St Andrews, UK; 2 University of Oxford, Oxford, UK; 3 Imperial College London, London, UK; 4 University of Plymouth, Plymouth, UK

**Keywords:** Curriculum design, Medical education, Medical humanities, Patient partners, Professional networks

## Abstract

Medical humanities teaching in UK medical schools has lacked cohesion, having developed opportunistically in different locations. Cohesion is necessary to develop an identifiable community of practice, but within that community there can be multiple readings of what ‘medical humanities’ are and how they may develop. This article details discussions held by medical humanities scholars teaching in UK medical schools at a workshop in January 2025 at the University of Oxford covering five key areas: the role of humanities scholars in medical schools, patients as partners in medical education, core curriculum teaching, intercalated teaching, and assessment. Our discussion highlights opportunities and challenges facing humanities teaching in UK medical schools today and calls for the creation of a community of medical humanities scholars working in UK medical education embracing diversity of opinion and practices. The article is specifically written as a synopsis of a brainstorming symposium.

## Introduction

The ‘medical humanities’ (used throughout in the plural) describe a disparate field characterised by interdisciplinarity. Broadly, the field considers how the arts, humanities, and qualitative social sciences impact upon medicine, including undergraduate, intercalated, and postgraduate medical education (Bleakley 2015, 2020, 2023). Where the introduction of arts and humanities interventions embraces health and social care, a more inclusive term– ‘health humanities’– is widely used (Crawford et al. 2020). The development of medical humanities in the UK can be traced to the birth of art therapy (Hill 1945) and has developed into three distinct streams. First, arts therapies became professionalised and are often employed as formal interventions in psychiatric settings; at the same time, use of the arts and crafts in the promotion of wellbeing amongst the public has developed as an ‘available’ and ‘democratic’ therapy (Crawford et al. 2020). Second, medicine is a topic for pure academic study– such as the history of medicine, medical ethics, literature and medical anthropology– that has little or no interest in application through medical education. In recent times this has been framed as ‘critical medical humanities’ which suggests an implicit entanglement between biomedicine and the arts and humanities rather than them acting as binary (Whitehead and Woods 2016). Third, and our interest in this paper, the medical humanities have developed as a pedagogical intervention across medical education, identified as a curriculum component in over half of UK medical schools (Revell and Blythe 2019, Howick et al. 2022), accruing an evidence base for efficacy (Bleakley 2015; Petrou et al. 2021).

However, development of medical humanities pedagogy has been haphazard and opportunistic rather than cohesive. Medical Humanities initiatives have largely focused on developing *syllabus* content, often working against a tide of resistance from core anatomy and biomedical science staff who protect ‘hard’ science content seen as more important to medical students than ‘soft’ arts, humanities, and qualitative social science input. Hard-won syllabus space is also often in the form of elective rather than compulsory components.

Such approaches have failed to build on successful initiatives such as the core, compulsory, and assessed programme developed at Peninsula Medical School (Universities of Exeter and Plymouth) in 2002 (Bleakley et al. 2006). The key innovation introduced at Peninsula has been ignored or misunderstood. Here, the focus was establishing a climate and culture of medicine through moulding the character of a *curriculum*, or course of study that produces an identity or character. Further, such curriculum shaping of identity was seen primarily as a product of radically transforming the nature of conventional anatomy and biomedical science input. Such input often resorts to a default instrumentalism or functionalism. However, through intensive staff development at Peninsula core science faculty engaged a wider range of values– the ethical, aesthetic, political, and transcendental (meaning)– in their teaching. Arts, humanities, and qualitative social science faculty shaped and guided this staff development programme (see Part 1 below).

We recognise that opportunities for shaping such radical curriculum process (rather than offering syllabus content) may not be an option at established medical schools and fighting for syllabus space may be the only way to keep the medical humanities flame burning. It is essential then for those in the field of medical humanities in medical education to share ideas and resources as a basis to lobbying medical schools and their parent organisational structures such as the General Medical Council. This requires developing cohesion as medical educators. Many medical humanities academics now involved with medical education do not view either medical humanities or medical education as their ‘home’ specialism, and they are often either the only or one of a few humanities staff employed by their medical school. This perhaps explains why there has been inconsistent engagement with the key national body, the Association for Medical and Health Humanities (AMHH), despite the organisation’s existence (and conferences) since 2000 and its close association with the British Medical Journal’s *Medical Humanities*– the key outlet for research.

What is missing is a sense of identity for disparate medical humanities scholars, researchers, and communities who teach as part of medical degrees. A more cohesive, professional community may be needed for those teaching humanities to medical students who together can tackle a critical challenge: securing a sustainable, robust role for humanities within UK medical education and expanding a research agenda. Such a community also needs to communicate better with the well-established research community of academic scholars who do not participate in medical education– such as historians of medicine and those who study medical ethics. Currently, there are as many roles for medical humanities in the medical curriculum as there are medical humanities scholars teaching in medical schools. This situation has developed organically as a relatively new part of UK medical education, and because medical humanities incorporate varied disciplinary backgrounds. How the field can cohere is crucial to ensuring that the medical humanities have an effective future within UK medical education. The unique challenges facing medical humanities scholars in medical schools include introducing medical students—typically well-trained in the sciences and positivist about its promises—to new ways of thinking about biomedical sciences; other disciplinary ways of thinking; adding the humanities to busy curricula in meaningful and productive ways intelligible to clinically/scientifically-trained colleagues; and advocating for the value of humanities in science, technology, engineering and mathematics (STEM)-focused environments. Without community, solutions to each of these complex issues will remain individual and ultimately institutionally fragile, subject to changing institutional priorities, staffing changes, and changing resource allocation.

To address some of these complex challenges, the authors organised an in-person workshop in January 2025, ‘Building a Network for Medical Humanities Scholars Teaching in UK Medical Schools’, hosted at Harris Manchester College, University of Oxford, funded by the University of Oxford’s Strategic Innovation Fund, John Fell Fund, and the School of Medicine at the University of St Andrews. The purpose of the workshop was to facilitate a series of discussions concerned with key issues that the authors identified in the teaching of medical humanities in UK medical schools. For this purpose, we invited medical humanities scholars who fit the description of ‘medical/health humanities trained academics who deliver teaching to medical students’ with a (not strictly enforced) limit of one member of teaching staff per university to ensure the workshop was representative of a broad range of UK medical schools. We identified several key individuals in advance from a search of UK medical school websites for evidence of medical humanities teaching. In addition to this, we made targeted invitations to clinically/scientifically trained colleagues, patient partners who had experience of medical humanities teaching, and former students of intercalated medical humanities courses. Thus, we created a targeted discussion that would go beyond persuasive recitals of the benefits of medical humanities to the uninitiated, to focus critically on today’s opportunities and challenges for medical humanities academics working in medical schools.

Dr Alan Bleakley, Emeritus Professor of Medical Education and Medical Humanities at Plymouth Peninsula Medical School, framed the day with an introductory lecture, followed by four workshops on current topics in the field. Below, we detail the significant outcomes of each session before providing a brief summary of overarching themes and questions. We aim to continue the conversations begun at the workshop in a wider academic setting. We hope, above all, to create a cohesive community for those teaching in UK medical schools. Our focus in this article is on areas for improvement that a cohesive community might tackle. However, the basis for many of the ideas and suggestions that were discussed is a shared sense of lost opportunity amongst our workshop participants that might now be regained: collectively we know that medical humanities have much to offer medical education, but we need fresh impetus. We also recognise the proliferation of medical/health humanities initiatives globally (e.g., Bleakley scussio^2020^; Lefève 2020).

The discussion that follows is premised on medical humanities inclusion in UK medical education as a research-based, or ‘best evidence’, initiative. The questions that remain centre around *how* medical humanities can be integrated into medical education; how medical humanities indeed are forms of medical pedagogy; and the roles of medical humanities scholars in that picture, rather than *if* medical humanities should be offered across curricula.

## Part 1: reflections on the medical humanities in medical education by Alan Bleakley

### The medical humanities in medical education

The role of medical humanities in medical education has largely developed since the late 1960s through trial and error. For example, bringing ethicists into medical schools makes sense if one takes into consideration the earliest motivation of medical humanities, which was to provide a corrective to the biomedical model; but how can musicians teach teamwork? Some areas of concern are obvious: medical students have traditionally learned anatomy through dissection and observation (looking) as a means of knowing what is going on under the skin. But doctors meet patients as moving bodies and so a better knowledge of surface and living anatomy can be gained through life drawing and drama. Drama and performance studies also investigate communication. Further, while patients’ records are largely quantitative (blood tests, blood pressure, urine examinations), how patients are known is qualitative. They are known through their histories, so narrative-based medicine is key to clinical work. In this way, literary scholars made their way into medical school faculties to work alongside clinicians, supplementing their work (Bleakley 2015).

The longstanding problem with this approach to the medical humanities is that it reinforces a traditional, and often unquestioned, division between sciences and arts. Medical humanities input into medical education has long been framed as compensatory for a biomedical sciences education (including anatomy) that is seen as the source for objectifying patients– reducing them to mechanics, symptoms, tests, and numbers. That biomedicine is reductive has some truth but is largely misguided. If biomedicine is learned as a lens through which patients are objectified, it is not biomedicine that is at fault, but rather the way that biomedicine is taught and learned. This is a pedagogical problem. Further, medicine in practice is informed by biomedical fact, but primarily deals with how patients experience their chemical flows and electrical pulses. What if we were to suspend the idea that the medical humanities act as compensation for a reductive biomedicine, concentrating rather on how we can enrich the teaching and learning of biomedicine that would lead medical students to not—characteristically—lose empathy and gain cynicism? What if medical students were taught to reveal, appreciate, explore, value, and employ in practice the intrinsic art and humanity of biomedicine? This would not mean that, in turn, arts and humanities input would be sidelined. Rather, arts and humanities—and qualitative social sciences—would be entangled with a rejuvenated biomedical science, where each would critically call out the others’ deficiencies. The issue of engaging medical education with medical humanities could then be framed as a staff development challenge: the shifting of values registers from the reductive, linear instrumental to complex aesthetic, ethical, political, and transcendental (meaning) registers.

### Medical education through the values Prism and extension to staff development

**Fig. 1:**
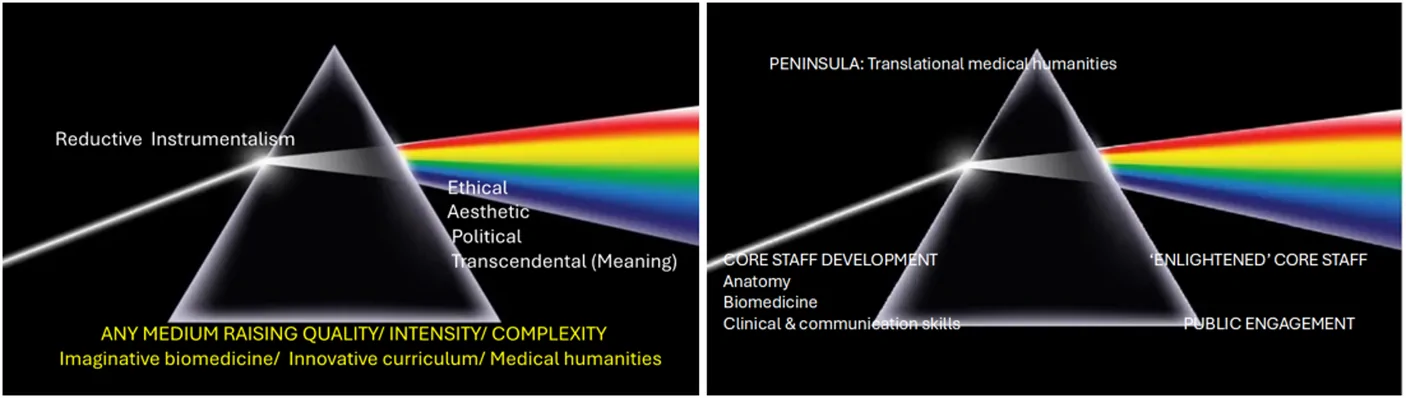
The Values Prism and application to staff development

This shift of values registers can be visualised as a bundle of white light (as instrumental reduction and compression) passing through a prism to create a rainbow of deeper values possibilities (Bleakley 2023, 2024) (Fig. 1). This describes a ‘translational’ mode of medical humanities. Here, the traditional medical humanities in medical education (as compensatory for a reductive or instrumental biomedicine) may be the medium for a values shift, but this can also be provided through pedagogic innovations and the re-imagining of anatomy and biomedical sciences. As noted earlier, at Peninsula Medical School, Professor Bleakley’s affiliated medical school, from 2002, core staff were engaged in staff development initiatives, emphasising pedagogical issues that transcended traditional ‘medical humanities’ approaches as compensatory for a rational and reductive biomedical science. This was contrasted, in a critically reflexive manner, to global medical humanities initiatives that seemed to us to be reductive and lacking in quality. Instead, the Peninsula School emphasised the notion of ‘curriculum’ (a course of study) as a variety of texts, already then introducing a literary lens of close reading to curriculum process models and their subsets of content or syllabi. Such curriculum texts include the political, ethical, aesthetic, transcendental, economic, historical, gendered, and autobiographical (engaging identity construction as a physician and ‘professional’) (Fig. 1). This challenged the dominant discourse of the reductive instrumental in medical education, advertised by a wholesale adoption of competences (rather than capabilities), information (rather than meaning), the literal (rather than the metaphorical), and the linear (rather than the complex).

## Workshop 1: the role of humanities scholars in medical schools today

Our first workshop began by considering the usefulness of ‘medical humanities’ as a role descriptor or job title: many participants felt that this term did not accurately reflect their subject-specific expertise and could in fact be unhelpful in obscuring precisely where their strengths lay. For example, within the remit of ‘medical humanities’ posts, we have found ourselves called upon to discuss ethical and legal questions beyond our specific areas of expertise.

The difficulty of defining the boundaries of our own roles and then identities was mirrored in our discussion of what exactly medical humanities offers to medical education– a question that has been extensively debated in the existing literature (Chiavaroli 2017; Colvin 2017, Gordon 2005; Mann 2017). Our focus in the workshop, however, was on how participants conceptualised their role within, and relationship with, their specific institutions. One participant suggested that a focus on transferable skills was more helpful than the study of specific subjects or ‘trends’ in medical humanities, and that there was in fact some value in ‘medical humanities’ being a vague descriptor. Several workshop participants suggested that medical humanities scholars needed to be involved in curriculum design and planning, albeit noting this was often difficult to achieve in large medical faculties. Another participant suggested that part of the role of medical humanities scholars was to ensure that humanities teaching delivered by clinical or scientific colleagues was ‘done well’. The need to avoid the humanities being perceived simply as ‘serving’ medicine (Wachtler et al. 2006; p. 16) was a common theme throughout our conversations. Mostly, this ‘handmaiden’ role has been eclipsed by the medical humanities acting as a ‘critical friend’ to biomedicine.

Narrative medicine is a key component of medical humanities (Schleifer and Vannatta 2013) and several participants also highlighted the value of stories in teaching, and as a means to engage students. One contributor expressed caution about stories where time-based narrative medicine often excludes space-based poetry (Bleakley and Neilson 2022).There was a general consensus that medical humanities teaching offered a space where stories could not only be valuable as teaching aids, but where students were able to tell their *own* stories (about experiences in medical education, for example) for the first time. Another participant noted that we should not be too quick to dismiss stories as ‘dangerous’, for example where they become confessional: if something is considered ‘dangerous’ then that speaks to its power. Stories were described as a way of helping students to appreciate their own position in the longer history of medicine and culture, as well as a means of highlighting how medical knowledge is ‘constructed’, rarely certain, and historically contingent.

Another common theme that emerged was the structural and institutional challenges each of us had to navigate in exploring or implementing change and feeling comfortable enough to ‘stick [our] head above the parapet’ (Blease 2016; p. 108). These challenges varied based on factors such as institutional funding and staffing. Administrative staff support for a humanities course or module was a given in one institution, for example, but entirely absent in another. The confidence and ability of humanities staff to make suggestions about medical teaching was directly affected by such variables. This was compounded by the sense that medical education was strongly hierarchical, with humanities often occupying the bottom rung of the ladder, filling in ‘gaps’ in curricula after core medical teaching had been scheduled in line with Bleakley and Marshall’s (2013) ‘weak inclusion’ model. This could lead to a sense of disenfranchisement among humanities staff. For those staff who were the only humanities scholar in their department, it was difficult to know where to start in terms of making meaningful suggestions for change, while also being mindful of their own potentially increasing workload. Their community of practice was more likely to be constituted by medical humanities practitioners such as those attending this workshop, rather than their anatomy or biomedical science colleagues within schools.

A realistic starting point for many participants was the idea of encouraging and supporting one another to ‘advertise’ our subject-specific expertise to clinical colleagues and medical faculty more broadly. While some have argued that the medical humanities should ‘avoid the temptation to become highly professionalised and expert dominated’ (Pattison 2003; p. 34), we contend that it is precisely the expertise of its practitioners that should be recognised, sought out, utilised, and celebrated. Jones et al. (2015) recommend looking out for ‘topics that other courses are struggling to cover’, and Steere-Williams et al. (2023) encourage historians of medicine (for example) to actively seek out leadership positions or take part in discussions about curriculum reform in their institutions. These are valuable suggestions. But what many workshop participants craved was practical advice, examples, and direct support about how to go about matters in their own *specific* situations– which were highly idiosyncratic and often did not match the published literature. The opportunity to discuss ‘the way problems arise and why they arise’ (Wachtler et al. 2006; p. 16) was clearly valued by workshop participants. This sense of impotence or lack of power can be countered by encouraging junior staff to seek positions on curriculum planning groups and other decision-making bodies.

Despite the practical challenges to its incorporation into medical curricula, the humanities clearly retain an important position within UK medical schools. Students at many universities study Student Selected Components (SSCs), elective modules, or humanities-based intercalated degrees that allow them to pursue their interest in humanities subjects beyond the bounds of their STEM degrees; others experience humanities as part of the core medicine curriculum. Such provision needs to be scoped, and a research project doing so is currently in hand.

## Workshop 2: cross-disciplinary collaboration and patients as partners

The topic was introduced with a key question: ‘Have we forgotten someone?’

Medical humanities in medical education have often been conceived of as tools for enabling conversations about—or building up—doctors’ empathic capacity for their patients. As described above, this compensatory model of medical humanities, which views empathy as a remedy for scientific reductionism, has been critiqued on the grounds that a better approach would be to fundamentally transform the pedagogy of biomedicine. Ideally this would be articulated through a medical curriculum where the humanity and totality of the patient is intrinsically centred and clinical encounters understood as an entanglement of identities rather than replications of a clear-cut binary between doctor and patient. As Martyn Evans has written:

The doctor and the patient tread two tracks across a world that is not by any means fully mapped out. Wherever their tracks intersect, the way in which they understand one another is moulded by their different pasts and futures. These are what fix the images and metaphors each will use, and they bring together two uniquely embodied experiences, each of which has somehow to make sense of and respond to the other. (Evans 2002)

Strikingly, patients themselves have not always been directly involved in these initiatives. One example would be the widespread use of simulated patients (e.g. actors trained to replicate the experiences of patients) in communication skills sessions. While there may be a fair rationale for replacing patients with actors, for example to ensure they are protected from difficult or uncomfortable situations, as Dylan Mulvin articulates in his history of the standardised patient, the attempt to standardise the irregular rhythms of patient experience can also be contested. This is because any notion of a ‘standard’ patients is enveloped in a perception of the ‘normal’ body — and indeed even a ‘normal’ diseased body. Thus, Mulvin argues that ‘what we see in this distillation of the standardized patient program is the way that normalcy is an imagined canvas for abnormality’ (Mulvin 2021).

The contention of this workshop, then, was that patient partnership is essential and readily achieved by working in direct and consistent collaboration with those who identify via lived experience of an illness or condition. The chairs described an initiative at Oxford University Medical School where patients with various neurological conditions co-lead sessions with senior clinicians. Such sessions give students the opportunity to learn about those aspects of conditions not covered by textbooks, for example the difficulty of turning over in bed for some patients with Parkinson’s; and receive, in real time, feedback from patients about practitioners’ clinical and communication skills.

The Oxford programme also includes patient involvement in humanities-based sessions. The ‘storytelling’ session is compulsory for fifth-year students, offering a blend of introductory concepts to narrative medicine, scenario work (where students are asked how they would communicate a broken MRI scanner to colleagues and patients), and an opportunity for students to ask patients questions, allowing both to explore ideas collaboratively (one participant, a clinician, noted, for example, the disjunction between the positive connotations of ‘progressive’, until it is connected to chronic disease for example). Another session sees students visit Oxford’s Ashmolean Museum to facilitate conversations about death and dying at seven ‘stations’ around the Museum, where they are also able to speak to the very patients who have helped design the sessions (Harris et al. 2024).

The current work of the programme is to expand the patient’s role in medical education, with an eye on local context and adaptability. Health inequalities in the local area are a key consideration, namely: how does inequality impact the stories told and which patient voices are heard?

The value of patient partnership was recognised by workshop participants in various ways: the honesty of the patient and doctor were key in helping students to recognise the limits of modern medicine, and to accept the idea that healing need not involve cure, as well as discussing bad experiences of diagnosis. One participant praised the programme as a genuine example of ‘participatory research’ and asked if there were any ethical insights, such as the payment of patients for their time (in this case, patients are paid). Clinicians are involved throughout to check on patient welfare, but it was also noted that patients are employed, trained, and are co-authors on published outputs. This training may also include dealing with difficult situations, such as encountering workshop participants who have loved ones with the same neurological conditions. The issue of ‘triggering’ students was raised; it was discussed that students at Oxford are given support but it was also emphasised that, in a professional context, their work life would involve dealing with personal triggers and part of the rationale for having conversations before graduation—and within a humanities rather than clinical space—was to help build skills to prevent these impinging upon patient care.

One participant noted that in their local area they had found Patient Partner Involvement (PPI) groups to be very interested in medical education, which opened another potential avenue for collaboration. Another said that such initiatives ideally need to take place throughout medical education, both at undergraduate and postgraduate level. At one institution, a student-selected component (SSC) was available where students can visit therapy initiatives at e.g. hospices, and work alongside patients without ‘interrogating’ them in the way that they might do in an Objective Structured Clinical Examination (OSCE). It was noted that it was sometimes hard to motivate patients to do something ‘extra’ that would assist medical education, and it was crucial to emphasise the value of patient insight to encourage this. One participant suggested that a performance element might be a helpful way to expand the reach, and awareness of, the Expert Patient Tutor (EPT) programme.

Then followed two parallel workshops tackling teaching the humanities in the core medical curriculum and intercalated degrees respectively.

## Workshop 3a: teaching the humanities to medical students in the core medical curriculum

We began the session by considering the General Medical Council’s (GMC 2018) *Outcomes for Graduates*. This is the central guiding document for UK medical schools as it ‘sets out what newly qualified doctors, from all medical schools who award UK primary medical qualifications, must know and be able to do’. It thus acts as a blueprint for the core curriculum as well as a framework by which medical schools are regulated by the GMC (ibid., p. 2). *Outcomes* includes specific guidance that ethics and law and sociology must be taught, so incorporates medical humanities defined beyond arts/humanities to embrace the social sciences, although how this is realised is at the discretion of individual medical schools. Several individuals at the workshop were employed specifically to teach such content in the core curriculum (some on education track contracts). In other schools, these aspects of the course were taught by clinically/scientifically trained colleagues with interests in those areas.

Clearly, where *Outcomes* align with medical humanities, there is an opportunity to advocate for their inclusion in the core curriculum. Yet, throughout our discussion there was a tension identified between the often-acknowledged value of medical humanities and the reluctance to make space for this within the core curriculum beyond where it is specifically blueprinted. With that in mind, we discussed how medical humanities can improve aspects of the core curriculum whilst adhering to the GMC’s guidelines. Several participants provided examples of where a ‘medical humanities first’ approach to teaching naturally covered several GMC core competencies such as being a good learner and teacher, being aware of one’s own health and wellbeing needs, tackling discrimination, and dealing with uncertainty in practice. For example, a key outcome of medical humanities teaching is to promote tolerance of ambiguity (Bleakley and Marshall 2013). Participants also discussed the potential to extend beyond these important competencies and into the ‘hard science’ parts of the curriculum: how might we be able to incorporate medical humanities in teaching on cell structure, for example?

This question raised a crucial issue: the perception of medical humanities methods—typically qualitative—as less rigorous than those typically used in science. There were concerns that as well as closing off assessment opportunities in the core curriculum, such attitudes also fed into perceptions of medical humanities teaching more generally: that it is viewed as less rigorous by some colleagues in the medical school environment. Participants discussed difficulties that they had encountered in medical schools in relation to their medical humanities teaching. These included timetable clashes that meant medical humanities teaching time was essentially pushed out by other ‘more important’ parts of the course and the difficulties encountered in changing aspects of the core curriculum, as its overstuffed nature meant that there was an inbuilt barrier to any new content that required a lot of work to overcome, creating inflexibility and inertia. Related to some of these issues, it was emphasised that the attitude at the top of medical schools is often critical towards the success of medical humanities within them. At times, issues that participants have encountered have been down to individuals ‘who don’t get it’. Conversely, where we have ‘allies’ in medical schools, opportunities increase. Here, we meet again the tension between a curriculum (process) approach in which a range of values beyond the instrumental are inherent, as against a syllabus/syllabi (content) approach in which information tends to displace meaning.

How to increase opportunities so that medical humanities scholars are less reliant on others’ ‘buy in’ and institutional politics then became a key theme. A question was asked if there was value in attempting to better quantify the success of our teaching methods, and evidencing how we meet learning objectives effectively? This would potentially be more recognisable to bodies such as the GMC. Another idea floated was of the creation of a ‘core curriculum’ document that would detail how GMC *Outcomes for Graduates* can be met by medical humanities teaching, as sociologists have created in relation to sociology teaching in the medical curriculum (Collett et al. 2016). If an agreed curriculum could be created it might be a powerful tool to influence decision makers formulating the medical curriculum. In local medical school settings, membership of internal committees concerned with ongoing issues like curriculum and assessment development, and the opportunities presented by co-teaching, were also discussed. It was acknowledged that this was labour-intensive work but work that had potential for impact. In particular, the opportunity to build multidisciplinary learning into the curriculum throughout, incorporating medical humanities but also other specialisms like nursing and psychology (where available), was argued to be a potentially powerful way to make medical humanities appealing to medical students. If students are used to dealing with multiple disciplines from day one of their degree, they come to think that ‘this is what we do in medical school’, widening their horizons.

It was noted that many of our clinical/scientific colleagues already incorporate aspects of literature, history, ethics, and other humanities content within their teaching. This could present an opportunity for medical humanities staff to train their colleagues, thereby introducing structure and rigour to such material. Some need for caution was expressed regarding staff education by participants, however, as it may create the impression that humanities can be learned via one or two discrete training sessions which could in turn undermine the role of permanent humanities staff in medical schools. At the same time, entering the students’ world, for example by attending ward rounds, was highlighted as an opportunity to develop medical humanities scholars teaching.

One way that we considered how medical humanities is taught in the core curriculum was via student selected components (SSCs). As the name indicates, students have a choice over the component they take, with the course of study typically taking place in a specified ‘block’ of time, usually around four or five weeks. They can be arranged by medical schools at any point in the medical degree. Our participants’ experiences of these ranged from running options that competed with ‘more fashionable’ choices such as sports medicine, to a variety of medical humanities options being the only ones available for students to choose. Whilst there were clear advantages to allowing flexibility of choice for students—only those interested would take them, making the likelihood they would appreciate it higher—the pitfall was that potentially those students who most needed to learn about alternative approaches would be unlikely to take the component. Where it was compulsory to take one of several options of selected components, there were significant opportunities to broaden students’ horizons and enhance their degree experience. However, issues with reluctant students were mentioned. In a single centre study at Imperial College London (6-year Bachelor of Medicine, Bachelor of Surgery (MBBS) course) from 109 responses, students felt strongly that humanities subjects should not be assessed (71%) but no consensus was reached on whether they should be elective or not (Petrou et al. 2021).

The group also drew an important contrast between SSC inclusion in the early years (1 and 2) of the medical course and later years of the course (e.g. years 4 and 5). In early years, students were viewed as very keen to engage—there was ‘buy in because this was what you did at medical school’ (alluding to the earlier discussion of multidisciplinarity in the curriculum)—but it was noted that students sometimes had little to reflect upon as their clinical work experience in early years is limited. By contrast, students taking SSCs in later years were viewed as having more to reflect on, but as being more cynical about the value of diverse approaches. Related to this, one contributor suggested that medical humanities can also be bad for students: it can be boring and disruptive of their studies, for example. Yet disruption may also benefit students: rather than being comforting the arts can challenge habitual routines and taken-for-granted values. Much talk around medical humanities in medical education fails to mobilise the disruptive qualities of the arts, for example by encouraging students to take a more critical approach to medical culture.

## Workshop 3b: teaching the humanities to medical students in intercalated degrees

In their survey of medical humanities teaching in the US, UK, and Canada, Howick et al. (2022) did not include intercalated degrees in their definition of ‘medical humanities courses’, viewing them as ‘additional, separate, degrees’ (p. 88). We include them here as they form a significant part of the medical humanities offering at several UK medical schools. The point at which students intercalate varies between institutions, and it was noted that teaching was very dependent on institutional resources. An intercalated degree at one institution ran with a maximum of five students, for example, while another regularly ran with between 20–25 students. Not all medical schools require students to do an intercalated degree, which dramatically affects recruitment numbers across institutions. More pertinent to continued recruitment to intercalated degrees is the fact that these degrees no longer contribute towards the Foundation Programme Application System (FPAS), which allocates medical students to foundation schools and jobs. Previously, students were awarded an extra FPAS point for undertaking an intercalated degree, plus additional points for a first-class result. This was changed in 2024 to level the playing field, as intercalated degrees were often the preserve of wealthier students able to afford an additional year of study.

The structure and character of intercalated degrees was very variable between institutions. At one, it was a full-time, year-long course with a dedicated teaching team who crafted original content spanning a variety of humanities topics. At another, students chose modules from elsewhere in the university and attended classes alongside students doing degrees in those disciplines. While the latter was a result of limited resources more so than design, its value was nevertheless recognised by workshop participants: medical students worked alongside their peers in other disciplines in a way that allowed them to see the value of the humanities for its own sake, and witness humanities scholars and students ‘at work’.

Challenges identified by workshop participants included ‘stigma’ about medical humanities from students or clinical staff, especially at institutions where intercalated degrees are compulsory parts of the medical degree. Here, humanities options were often viewed as less ‘relevant’ than more traditional intercalation topics such as pharmacology or respiratory medicine. Students often felt they had to ‘justify’ a humanities intercalation to their peers (and, potentially, to future employers). As well as pedagogical concerns about presenting a ‘watered down’ version of humanities topics to medical students on intercalated degrees, the intensive nature of these degrees often presented additional administrative challenges for teaching staff. The availability of sufficiently trained second markers for assessments, for example, could be a major problem, especially when there were not many humanities staff within the medical school or faculty. One workshop participant described liaising with a second marker for humanities essays who had marked first-class student work in the third-class category, due to not being familiar with humanities conventions such as incorporating direct quotations from secondary sources to back up points. Such differences in approach took a great deal of staff time (on both sides) to resolve amicably, but could, it was noted, be remedied by providing more training or guidance to clinical colleagues about humanities conventions.

On a more positive note, it was recognised that intercalated degrees tend to be the point at which many medical students realise their need to develop better professional skills, such as time management and organising workload. With learning in intercalated humanities degrees less directed, and students freer to organise their own reading and research, some found this a difficult adjustment. The skills offered by humanities education were noted to be of great value here, as well as for future professional practice: organising one’s own research for qualitative assessment, refining writing skills, and providing creative solutions to problems. The greater focus on essay-writing in many intercalated degree programmes may also be the first point at which students become aware of any Specific Learning Difficulties (SpLDs) such as dyslexia, which are less obviously detected in earlier year assessments such as multiple-choice exams.

With intercalated degrees in the humanities offering greater opportunities for students to expand their disciplinary horizons, there were several suggestions for enhancing teaching at this level to involve other stakeholders. Making connections with museums and archives to explore object-based teaching, for example, was an approach that several participants described at their own institutions, in the form of guided tours of exhibitions as well as having students design and run their own tours of heritage sites. The possibility of week-long ‘taster’ courses for medical humanities was another suggestion, with one participant noting that a similar endeavour had once been run by the Wellcome Collection. Again, though, the pressures of the medical student’s curriculum had to be borne in mind, and how many students would take up such an opportunity; an alternative might be for humanities staff to run taster days or lectures to provide both students and staff within their faculties with the opportunity to learn more.

## Workshop 4: assessing the humanities in medical curricula: challenges and opportunities

The final workshop on assessment provided an opportunity for participants to share tried-and-tested methods from their own practice, as well as potentially innovative ways forward in forming creative but curriculum-relevant modes of assessment in medical humanities. This was aimed at tackling the well-established phenomenon of assessment-driven learning in students (Wormald et al. 2009). Indeed, a perceived lack of engagement of students in humanities teaching may be a reflection of the knowledge-based summative assessments necessitated by medical school, with humanities being difficult (though not impossible) to assess in the traditional modes of Multiple-Choice Question (MCQ) exams and Short Written Answer/Short Answer Question (SWA/SAQ) exams. However, the diverse nature of medical humanities was felt to provide opportunities for improving assessment of medical students, where facts acquired from medical textbooks can quickly become outdated. One aim presented at the day was to ensure that ‘tomorrow’s doctors’ develop a professional identity that prepares them for the complexities, changes, and ambiguities they will encounter throughout their careers at the core of which is tolerance of ambiguity and uncertainty. Indeed, Pfeiffer et al. (2016) review the notion that medical humanities programmes have turned to scenario-based assessments to demonstrate competence in professional identity formation, with problem-based learning (PBL) approaches garnering a less reductionist, more interpretative approach to the material at hand (Goodwin and Machin 2016).

A key aim of the session was therefore to collate a broad range of examples from participants of different kinds of assessment, summarised in the table below (Table 1). Current examples of assessment described in this workshop included the ‘traditional’ such as MCQs on humanities topics, on themes such as narrative medicine and philosophy. One participant noted that this constituted around 5–10% of exam content in their institution. In a very different vein, an activity in which students delivered a tour to the public at a site of medical heritage was discussed. This is assessed for strength in negotiating uncertainty and communication skills. Other forms of assessment mentioned included podcasts, creating Wikipedia pages, and creative responses such as a piece of art or dramatic performance. In one case, it was noted that the assessment was not of the quality of the artefact (painting, music, poetry, etc.) itself, but in the reflective process following it– asking the question ‘how has making the artefact enabled me to look differently at a clinical issue or a patient?’ This is assessed via a short essay and presentation. The pedagogical logic here is creating a ‘second stimulus’ (arts and humanities input) to provoke a ‘defamiliarisation’ or ‘thinking otherwise’ (Bleakley and Neilson 2022).

Several challenges were noted with assessment, perhaps most pressingly the need to ensure ‘assessment enhanced medical humanities’ links to the GMC’s *Outcomes for Graduates*. It was also noted that assessment for pre-clinical students may need to be different than for those in their clinical years. Colleagues from one institution gave the example of authoring clinical case reports as a potential assessment method for the latter students, a way of both preparing students for one of the key tasks of the profession, as well as highlighting the necessity of good quality writing for the sake of good communication.

**Table 1:** Summary of some of the methods of assessment discussed

Method of assessment	Description
Multiple Choice Questions (MCQs)	A short introductory vignette followed by a question that has five potential answers (written as options a through to e), one of which is correct. The student must select only one answer.
Short Written Answers/ Short Answer Questions (SWA/SAQs)	Students are expected to provide short written answers to set questions. The marks to be awarded are stated as part of the question. For example, a question worth two marks would require two distinct pieces of information written by the student.
Creative outputs	There are two elements to this: (1) the production of a piece; (2) reflection upon how the making of the piece has informed the student’s medical education. This reflection can be written or presented or both, with marks being awarded only for the reflective portion of the assessment. Examples discussed included dramatic performances, graphic novels, and sculptures.
Seminar/presentation	Students produce these independently or collaboratively in small groups. They are marked on teamwork (if relevant), content and responses to questions. Some take the form of poster presentations, with the posters also marked for content, design, etc.
Dissertations/essays	These allow for more in-depth analysis of concepts and ideas. In large cohorts, the time taken to mark these can be prohibitive as Faculty able (and available) to mark (and double mark) the scripts is a rate limiting factor.
Podcasts/factual vignette/Wiki	Podcasts test the ability to synthesize and present complex information for a general audience. Similarly, a Wiki demonstrates the ability to present information in a written format that is not only easily understandable but factually correct.
Book reviews	This enables the assessment of critical appraisal skills and could also be of use in a career whereby this may be necessary for journal contributions.
Group tour	Designing and delivering a group tour for visitors at a site of medical heritage, including producing a leaflet and reflecting on visitor feedback. These were dependent on cultivating working relationships with heritage partners.

## Summary of key points

At the outset of this article, we called for cohesion across medical humanities provision in medical education. In terms of pedagogic approaches, the field is multifaceted and shaped by differing interests, enthusiasms, and expertise reflected as a current lack of coherence. However, cohesion– the identification with a community of practitioners– is a prerequisite for coherence. Indeed, coherence may not be necessary– a field of inquiry can be represented by differing approaches. But, cohesion across practitioners means that we can discuss such differences amicably and productively. To facilitate this discussion, the key take-away points of the day’s discussion are summarised below:

*Medical humanities have a lot to offer medical education.* Throughout the day, participants provided ample evidence of the huge range of benefits offered by incorporating medical humanities into medical education, complementing the substantial literature on the subject.

*Medical schools are amenable to incorporating medical humanities into the curriculum with conditions.* The myriad and diverse examples that participants gave of how medical humanities has been included as part of the educational offer of their respective medical schools, underlines that they will include medical humanities in medical curricula. Beyond simply showing that medical humanities have become increasingly established in UK medical education, this fact demonstrates the success the field has had in articulating arguments for its incorporation. Using evidence and learning from past successes is crucial to establishing the field more fully in UK medical education.

*Medical humanities are institutionally fragile in medical schools.* Often medical humanities are reliant on one or two individuals to develop and run bespoke offerings for their schools. A small institutional footprint can mean that the content/module/programme that is run is reliant on those individuals for its continuation and staffing, with changes due to e.g., staff retirement or changes of Dean potentially spelling the end of well-developed programmes as an ‘easy’ cut. Whilst there are many colleagues in science and medicine who ‘get it’, there was a feeling that medical humanities scholars are over-reliant on ‘allyship’ from interested colleagues in a way that other parts of the medical curriculum are not.

*Strategic thinking about our ‘offer’ to medicine is necessary.* As medical humanities scholars teaching in UK medical schools are dispersed across diverse institutions, and because they are representatives of a multidisciplinary field, there is an acute need to think strategically about our common challenges, opportunities, aims, and goals. Sharing best practice and ideas is an important first step, but going beyond that to consider how our work as educators relates to the broader context of UK medical education is a vital next step and requires strategic thinking. We need to consider what it is that we want to prioritise and how to obtain that. This may include: developing a ‘core curriculum’ that acts to unify what medical humanities is seen to offer medical schools; campaigning for key medical education bodies such as the Medical Schools Council and the General Medical Council to improve the medical humanities offer by UK medical schools nationwide, or the development of specific medical humanities outcomes in the GMC’s *Outcomes for Graduates* beyond the existing ethics and sociology outcomes. The potential for medical humanities to become fully established in UK medical schools is there, but thinking carefully about both what we want to prioritise and how we go about facilitating change is important.

*Digital resources need to be improved*. There was a felt to be a general lack of digital resources relevant to those teaching in UK medical schools. This covered both online teaching resources, as well as advertisement of teaching and research opportunities in a centrally recognised location that those teaching at medical schools might utilise to meet and collaborate. The difficulty of finding basic information that might aid those teaching in medical schools was seen as a significant barrier to creating sector-wide collaboration and coherent policy.

*Potential for patient partner engagement.* Key legislation mandates a commitment to patient and public involvement and engagement (PPIE) in healthcare (Health and Social Care Act [2012] and the NHS Constitution). Given that a major role of the humanities in medical education is the formation of professional identity then it follows that the patient should be front and centre of delivery of teaching and, some may argue, assessment. One of the shortfalls of PPIE can be considering those who are not in the room. The humanities enable outreach to those hard-to-reach patients, through knowledge of local histories and an understanding of the richness of patients’ stories. An argument can therefore be made that the humanities not only enable formation of a professional identity but are also key in ensuring that the identity that is formed is culturally sensitive and inclusive.

*Staff continuing education as an opportunity.* Our clinical/scientific colleagues often already reach for the medical humanities in their teaching via history, metaphor, literature and so on, but on an ad hoc basis. As a result, there is an opportunity for medical humanities scholars to utilise staff training days and other educational mechanisms to develop the rigour of these uses, leading to more nuanced use of medical humanities across the curriculum. However, this comes with the caveat that such education should not be viewed as a replacement for medical humanities scholars themselves. Some of the challenges discussed in the workshops were ones that could be relatively easily resolved with more targeted education and/or information sharing. Putting humanities and clinical colleagues into more meaningful, practical, dialogue about issues such as differences in assessment expectations between the humanities and medicine, or concepts of ‘bias’, could lead to cultural change.

*Tension between education and research*. Whilst many of those teaching in medical schools are based primarily in humanities departments, several participants discussed being employed on education-only contracts. In these cases, individuals had been hired in part because of their experience in research but then found they had much reduced time (and responsibility) for their research. While this is not uncommon for science/clinical staff in medical schools—many are not research active—it was felt to be a significant limitation for humanities scholars, as a key part of humanities scholars’ working lives is participation in (inter)disciplinary discourse(s).

*Research support.* For those humanities scholars employed directly by medical schools and engaged in research within their own discipline—and especially those on teaching-only contracts—it was often difficult, if not impossible, to secure either funding or research leave. Institutional support for research tended to be available only for projects that addressed medical education directly, with some STEM-focused institutions lacking any funding infrastructure that fitted with humanities scholars’ discipline-specific needs. Yet, many of these same scholars incorporate their research into their teaching offerings, in line with the pedagogical concept of research-led teaching.

## Conclusion and next steps

The workshop was facilitated to begin the creation of a cohesive community of medical humanities scholars teaching in UK medical schools that may tolerate some lack of coherence across values, theory, and practices– perhaps a positive sign of diversity. By meeting to discuss shared experiences, the community-building process has begun, but needs further work to make it sustainable. To that purpose, we have created a JISC mailing list for medical humanities scholars teaching in UK medical schools (TeachMedHums) to better connect individuals, are currently surveying medical humanities scholars employed to teach in UK medical schools on their roles to better understand the needs of those in these positions, and have further digital and in-person events planned for the near future to continue to meet and discuss the opportunities and challenges facing medical humanities scholars in UK medical schools. Also, we are networking with a medical humanities forum from St George’s Hospital, London and with the AMHH.

Medical humanities scholars teaching in UK medical schools occupy a unique position, offering valuable multi-disciplinary perspectives that can enhance medical education. But their unique position also requires them to navigate the ideological and administrative challenges that this role can bring. Our workshop identified some key challenges that participants faced in common in their roles, as well as areas ripe for further development. We noted a burgeoning literature within medical humanities globally and here we have just scratched the surface of that resource. Most importantly, we share a common identity of medical humanities *practitioners* who draw on relevant scholarship as well as seeking to add to it for the benefit of medical education.

## Data Availability

No datasets were generated or analysed during the current study.
